# Development of a Comprehensive Food Data Citation Standard: A Surprising Gap in the Nutrition Research Literature

**DOI:** 10.1016/j.cdnut.2023.102048

**Published:** 2023-11-24

**Authors:** Shavawn Forester, Emily Jennings-Dobbs, Britt Burton-Freeman

**Affiliations:** 1 Nutrient Institute, a 501(c)(3) not-for-profit organization, Reno, NV, United States; 2 Department of Food Science and Nutrition, Illinois Institute of Technology, Chicago, IL, United States

**Keywords:** food and nutrient composition data, data quality, precision nutrition, data citation, citation guidelines, data sharing, nutrition research, scientific integrity

## Abstract

Currently, there is no standard for the citation of food composition data. This leads to the questions: how are food and nutrient data cited in research papers, and are they presented in a way that allows studies to be reproduced? To answer these questions, we performed a review of the literature and quantified the accuracy and completeness of data citations from publications (January to December 2020) in the top 5 nutrition journals as ranked by the Scimago Journal Rankings. We then performed a review of citation guidelines currently in place in other disciplines. Similar to the requirement of completing the Preferred Reporting Items for Systematic Reviews and Meta-Analyses checklist for systematic reviews, we have developed a comprehensive data citation checklist, the Comprehensive Food Data Citation (CFDC) checklist. The CFDC checklist was developed through a benchmarking assessment against established data citation standards. Its purpose is to establish a standardized, best-practice approach for reporting food composition data. The CFDC checklist has been designed to cater to both publishers and authors, ensuring consistency and accuracy in food composition data reporting. The CFDC checklist is also available as an interactive citation generator to facilitate the adoption of consistent and comprehensive citation of food composition data and is available at https://www.nutrientinstitute.org/cfdc. Despite general agreement that accurate data citation is paramount, this is the first citation standard specifically developed to capture food composition data. Because food composition data are the foundation of nutrition research, our proposed guidelines aim to provide the field with a much-needed foundation for acknowledging and sharing data in a way that fosters reproducibility.

## Introduction

Attempts to replicate research data have been largely unsuccessful in several fields of research. In the recently completed 8-y Reproducibility Project: Cancer Biology, less than half of preclinical research results were successfully reproduced from top journals such as *Nature*, *Science*, and *Cell* [1], because of unclear protocols and data missing from statistical analyses in the studies that were examined. Insufficient specification of study conditions, such as materials used, data retrieval, and analysis details, created ambiguous circumstances for reproducing data and drawing conclusions. In addition, the terms “reproducibility” and “replicability” are used differently across various scientific disciplines. Here, we refer to “reproducibility” as “instances in which the original researcher’s data and computer codes are used to regenerate the results,” and “replicability” as “instances in which a researcher collects new data to arrive at the same scientific findings as a previous study” [2].

Using standardized guidelines and checklists for data collection and analysis offers a practical remedy for addressing reproducibility and replicability problems—and, ideally, for preventing such problems before they can occur. In 2018, *Nature* published an editorial reviewing the benefits and efficacy of checklists for reproducibility in biomedical sciences and demonstrated increased confidence in results produced using such checklists [3]. The PRISMA guidelines and checklists are an example of a rigorous and successful data citation standard. The PRISMA guidelines and checklists were created in the 1980s to make data more transparent and complete so that systematic reviews could be compared [4,5]. The PRISMA standards have been broadly adopted and were updated in 2020 [6]. To reproduce published work, scientists must be able to access the original data, protocols, and key research materials and resources—including the specific databases used [[7], [8], [9]]. Because complete and accurate data citation is the key to reproducibility, many authoritative science policy bodies are calling for robust archiving and citation of primary research evidence [10]. Although journals encourage authors to cite underlying or relevant datasets in the manuscript and as a separate reference, and provide specifics on what data references should include, this encouragement is unfortunately not enforced. These inconsistencies and gaps in data citation practices underline the need for standardized guidelines and greater adherence to ensure that essential information is consistently provided in research publications [11].

The field of nutrition sciences encounters shared challenges in terms of reproducibility and replicability. Reproducibility challenges in nutrition sciences can be attributed to various factors, including the inherent variability of natural products, the variable or unknown composition of ingested food and beverage products, as well as the variability among individuals, which has been a focus of recent precision nutrition endeavors [12,13]. Another factor, perhaps overlooked previously, is variable database citing practices for food composition data. This exacerbates the already recognized inherent variability of foodstuffs, humans, and experimental conditions. The absence of robust and standardized citation of food composition data introduces potential variability impacting reproducibility efforts. This inherent lack of precision also poses a significant obstacle to fully realizing the potential benefits of precision nutrition initiatives, such as the NIH Nutrition for Precision Health initiative [9].

Citation guidelines for databases exist in other scientific fields; however, standards do not yet exist for citing food composition data. This study, therefore, aimed to first identify the current state of food and nutrient data citation and then to facilitate the development of a comprehensive food and nutrient data citation standard. For the first aim, we used a cross-sectional descriptive analysis to assess citation accuracy and completeness. For the second aim, we created a standardized checklist and interactive data citation generation tool using a set of citation criteria drawn from current data citation guidelines promoted in data science fields. The proposed Comprehensive Food Data Citation (CFDC) checklist is intended as a guide for authors and reviewers. Our goal is to provide the most comprehensive guidelines and interactive tool for standardizing citations for nutrient composition data, to support and advance nutrition research.

## Methods

### Literature search

A procedural document was created and followed step by step to achieve a systematic approach to collecting data. This procedure document can be provided by the corresponding author. Publications were identified through a search of articles published in the year 2020 in the top 5 nutrition research journals, as ranked by Scimago Journal Ranking (SJR) [14]. The most current full year at the time of the search, 2020, was selected to represent current use of food and nutrient data. We chose these respective metrics as proxies for the highest quality food composition data and citation standards and the most recent use of food composition data within the nutrition research community. The SJR ranking system was specifically chosen to focus on high-impact journals in the field of nutrition and dietetics. The 5 journals identified by SJR were as follows: *Annual Review of Nutrition*, *International Journal of Behavioral Nutrition and Physical Activity*, *American Journal of Clinical Nutrition*, *Advances in Nutrition*, and *Nutrition Reviews*.

The publication search was initiated in June 2021 within each journal’s search engine, filtering to the correct year/volume (2020). Qualifying publications were identified with a preliminary review of each article by title and abstract. Keywords were identified and used to identify qualifying articles. Titles and abstracts were screened in triplicate for keywords; any article containing one or more keyword(s) in the title and/or abstract was identified for further review. Keywords were “nutrient,” “nutrients,” “nutrient density,” “nutrient profiling,” “nutrient composition,” “diet quality,” “nutrient values,” “macronutrients,” “micronutrients,” “vitamins,” “minerals,” “diet patterns,” “diet,” “meals,” “snacks,” “drinks,” “food,” “nutritional aspects,” “nutrient content,” “nutrition content,” “nutrient timing,” “nutrition requirements,” “dietary requirements,” “dietary behaviors,” “food behaviors,” “database,” “nutrient database,” “food database,” “weight loss,” “food data,” “nutrient data,” “food composition,” and “composition data.”

Publications were further reviewed in detail by downloading and reading each in its entirety, including footnotes, tables, and figures. If supplemental data were identified as containing further information about food composition data, the supplemental data were also downloaded and reviewed. Publications that did not use food composition data were excluded.

### Citation identification and categorization

Publications identified in the manual literature search were subjected to a second review to identify all citations of food composition data. Food composition data used in a publication may be referenced in the text of the publication, in table footnotes, figure legends, the references section, or provided in supplementary material. Therefore, all mentions of food and nutrient data found within the text (descriptive citations) and formal citations found in the references section were recorded. If descriptive and formal citations were provided together, they were assessed as 2 parts of the same citation. Further classification was required to better evaluate where and how citations were handled. Predefined categories were defined as follows: *1*) unidentifiable source of food composition data—data source could not be identified from the information provided in the citation; *2*) not a source of food composition data—citation led to a source that did not provide food composition data (for example, Dietary Reference Intakes); *3*) conflicting source of food composition data—information provided by the descriptive (in-text) citation did not correspond with information provided by the formal citation (that is, different version, different source, etc.); and *4*) direct and identifiable sources of food composition data—the citation provided enough information to directly identify the source of food composition data. For those citations leading to identifiable sources, the title was documented as well as the year published or edition, Uniform Resource Locator (URL), and mention of any edits made to the data, which could include additions, deletions, and/or transformations.

The quality of a data citation can be measured by the components of the citation: a minimally complete citation provides just enough information so that the data used can be found. Therefore, to assess the current state of data citation quality, this analysis considered a citation to be accurate if it contained no conflicting information, and to be minimally complete enough for research reproducibly if it provided at least the title, year published or edition, URL, and any edits made.

### Data citation guideline benchmarking assessment and review

We conducted a search of publications reporting citation guidelines and citation scoring frameworks using Google on a Chrome web browser (date retrieved 4 August, 2022, location: Jersey City, NJ; Chrome web browser version 100.0.4896). Initially, this search was conducted on the PubMed search engine, but because of limited results, a standard Google search was selected as the most robust search strategy to find data citation guideline standards from a broad set of online locations. Search keywords included “citation scoring framework,” “data citation scoring,” “data citation scoring guidelines,” “assessment of data citations,” “citation guidelines,” “standards for data citation,” “data citation standards,” “guidelines for citing data,” “components of data citation,” and “data citation guidelines and phrases.”

Once the guidelines were collected, they were combined into spreadsheets for comparison. Each guideline provided a unique list of components, which constituted information required for a citation to be complete and facilitate reproducible research. These spreadsheets contained the title of the citation guideline, name of citation components (for example, title, version), and definitions associated with each component. Different guidelines had overlapping components. For example, the component “author” in one guideline might be referred to as “creator” or “Principal Investigator” in another guideline. To maintain all vital components while eliminating redundancy, we selected the most common name for a component based on corresponding definitions and grouped these analogous components under that most common name. All guideline components were included because each served a unique purpose. Once grouped, the components were formatted into a checklist with examples. These components provided the basis for a set of comprehensive guidelines for citing nutrient composition databases. We note that not all citation components are necessarily applicable to every citation. For example, data may not be accessible in a physical location (see Table 1, Formal Citation Component 7). However, each citation should include all relevant citation components.

**TABLE 1:** Comprehensive Food Data Citation (CFDC) checklist

	Citation component	Description	Example (USDA SR legacy)	
Formal citation components				
1	Author(s) of data cited	First name or initial, middle initial (if applicable), and last name of all authors	Haytowitz DB, Ahuja JK, Wu X, Somanchi M, Nickle M, Nguyen QA, Roseland JM, Williams JR, Patterson KY, Li Y, Pehrsson PR	□
2	Name of parent-series containing data cited (if applicable)	Full name of parent-series (not abbreviated)	FoodData Central	□
3	Formal name of data cited	Full name of data (not abbreviated)	United States Department of Agriculture National Nutrient Database for Standard Reference	□
4	Version of data cited	Release of the data, such as: Release #, Volume #, Version subtitle If multiple versions of the data were used, all versions must be provided	Legacy Release	□
5	Edition of data cited	Specific versioning information, such as: edition # and/or revision date If multiple editions of the data were used, all editions must be provided	Modified 2022 Jan 07	□
6	Resource type	Physical medium of the data, such as: Dataset, Text, Audio, Book, Journal Article, Newspaper Article, Website, or Magazine Article	Internet dataset	□
7	Physical location where the data can be accessed (if applicable)	Building name (if applicable), Street # Street Name, Building #/ Apt #, City, State, ZIP Code™	Beltsville, MD	□
8	Publisher(s) of the data cited	Organization Name(s) of publishers	Nutrient Data Laboratory, Beltsville Human Nutrition Research Center, ARS, USDA	□
9	Publication date	Year, month, and day data were published	2019 May 07	□
10	Access date	Year, month, and day data were accessed or procured	Accessed 2022 Nov 07	□
11	Global persistent identifier	String(s) of numbers, letters, and symbols that provides a permanent web address, such as: DOI, URL handle, archival resource keys, uniform resource names, and/or persistent uniform resource locator Multiple identifiers may be provided	https://data.nal.usda.gov/dataset/usda-national-nutrient-database-standard-reference-legacy-release identifier: 69ebc253-1869-4bf0-8471-b0c2fb5742f5	□
12	Person(s) or organization(s) responsible for funding the collection of the data (if applicable)	Organization Name(s) or full name(s) of individual funder(s)	Agricultural Research Service	□
Descriptive citation components				
13	Subset of data cited (if applicable)	Include all data identifiers, methods of joining data, and filters used	Filtered only to contain foods in the food group "Dairy and Egg Products", as specified by the "FD_GROUP" file	□
14	Edits made to the data cited (if applicable)	All modifications, no exceptions	All units were transformed from milligrams to grams; missing nutrient measure values were inputted from scientific literature, sources identified in supplemental tables	□
15	Code book or any other resource needed to interpret the data (if applicable)	If the code book was provided as part of the dataset, codebook citation is not necessary. If codebook was provided separate from the dataset, it must be accompanied by its own citation	Data dictionary available at https://data.nal.usda.gov/dataset/usda-national-nutrient-database-standard-reference-legacy-release	□

An inclusive checklist for food composition data citations. Formal citation components are required to locate data used. Descriptive citation components include how the data were used or modified by the author. Not all citation components are applicable to every citation.

## Results

### Literature search

**FIGURE 1:**
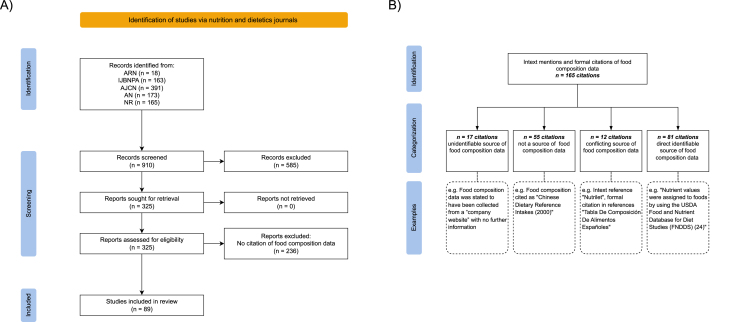
Manual literature search flowchart and data citation categorization. These flowcharts depict (A) an overview of the manual literature search and (B) the analysis and categorization of citations. Current food composition sources were identified, searched, and screened. Publications were selected based on the Scimago Journal Ranking (SJR) [14], the top 5 journals in the category nutrition and dietetics as ranked by SJR in May 2021, were *1*) the *American Journal of Clinical Nutrition* (AJCN), *2*) *Advances in Nutrition* (AN), *3*) A*nnual Review of Nutrition* (ARN), *4*) *International Journal of Behavioral Nutrition and Physical Activity* (IJBNPA), and *5*) *Nutrition Reviews* (NR). For each of the 5 journals, a publication search was performed using the search engine on the journal website, filtering for publications in the year 2020. The year 2020 was specified to identify the most current use of food composition data at the time of collection. Citations collected from publications were organized into categories defined in branching boxes; examples for each category follow in dotted lines.

After final review of publications (*n* = 910) (details provided in Figure 1A), 89 publications were identified as using food composition data and were included for further citation identification and categorization. Publication types were recorded as follows: Editorial (*n =* 2), Original Research Communications (*n =* 72), Perspective (*n =* 2), Research (*n =* 7), Review (*n =* 1), Special Article (*n =* 3), and Supplement Article (*n =* 2).

### Citation analysis

From among the 89 publications included for descriptive analysis of data citation, 165 data citations were identified; several publications included multiple unique citations.

Component details were captured for both formal and descriptive citations. Each formal citation and descriptive citation was evaluated as an independently valuable data reference because there was no consistency to what was provided in formal compared with descriptive citations. Components such as publisher and version could be found in both descriptive and formal citations. In one instance, a URL was provided in-text and no formal citation was provided. This lack of consistency made it difficult to determine how and where data were being cited.

These 165 citations were then assessed by category. Figure 1B provides a schematic of the citation analysis, category definitions, along with examples. Overall, 84 of the 165 (50.9%) citations for food and nutrient data, spanning the first 3 categories displayed in Figure 1B, did not provide accurate information and/or lacked information and thus could not be used to identify a food composition data source.

#### Unidentifiable citations

The first citation category, “unidentifiable” (*n* = 17), addressed citations that were unidentifiable because of incomplete information: 5 citations had no source identified in-text and no formal citation was provided; 3 citations had no source identified in-text and a formal citation was unrelated or too vague to identify a data source; and 9 citations had a vague or unidentifiable source identified in-text and the corresponding formal citation was also vague or not provided. For example, one citation stated in the text that food composition data were collected from a “company website” but provided no further information. Another example was when a source of food composition data were mentioned in-text so vaguely that it could not be found, such as “Star of Nutrition” software. And as a final example, a citation provided the nutrient composition (for example, total fat, carbohydrate, etc.) of a test meal muffin in supplemental material, yet provided no references to either food composition data or lab analysis.

#### Non-food composition data citations

The second citation category, “not a source” (*n =* 55), addressed citations that did not lead directly to food composition data. These citations were deemed inaccurate because they did not lead to the original source of food composition data used in the analysis. The majority (*n =* 23) led to large-scale dietary intake and health surveys such as the NHANES and the associated What We Eat in America. Another set of citations (*n =* 20) led to publications rather than citing the original source of food composition data itself. Additional citations (*n =* 6) led to sources of dietary standards and guidance such as Dietary Guidelines for Americans. Lastly, remaining citations (*n =* 6) led to food intake-type surveys such as food-frequency questionnaires.

#### Conflicting citations

The third citation category, “conflicting” (*n =* 12), addressed conflicting citations, instances in which both an in-text and corresponding formal citation were provided but contained contradictory and therefore inaccurate information. These included 2 citations in which in-text and corresponding formal references led to different versions or editions of the same data and 10 citations in which corresponding in-text and formal references identified different food composition data sources altogether. For example, an in-text citation for “Nutrilet” had a corresponding formal citation for “Table of Composition of Spanish Foods.”

#### Direct identifiable citations

The fourth citation category, “direct identifiable” (*n =* 81), comprised citations with enough information to identify the source of food composition data used. Although all 81 citations provided a title for the food composition data source utilized, no single citation provided all components required to be minimally complete enough for research reproducibility. Nearly 1 in 3 (28.4%) citations did not provide information on the edition of the data used. For example, the USDA National Nutrient Database for Standard Reference (SR) was cited 6 times for various release editions: 1 for SR26, 2 for SR27, 1 for SR28, 1 for the SR Legacy Release (USDA SR Legacy), and 1 with no edition specified. Each of those different versions of SR contains different information. A URL was provided for 19.8% (16 of 81) of included citations, of which only 12 URL links provided worked at the time of collection. In this sample, 8.6% of citations were accompanied by a description of modifications made to the data reported.

### Data citation guideline benchmarking analysis

Our benchmarking analysis identified 16 data citation frameworks across multiple disciplines, including International Association for Social Science Information Service and Technology [15], Earth Science Information Partners Data Citation Guidelines [16], and Data Citation Adequacy Index [17]. All citation components from each framework were documented, along with their descriptors. The descriptors were then used to consolidate all identified citation components from each framework (Supplemental Table 1) into one common, comprehensive set of non-redundant citation components (Table 1). This analysis identified 15 unique citing components among the 16 frameworks. Table 1 displays the common citation components identified that form the proposed CFDC citation checklist.

### Developing the CFDC citation generator

The CFDC citation checklist includes the 15 unique citing components identified in the benchmarking analysis. In addition to the citation components, the CFDC citation checklist provides additional descriptions for each component and corresponding examples. The examples draw from hypothetical use of USDA SR Legacy food composition data [18].

**FIGURE 2:**
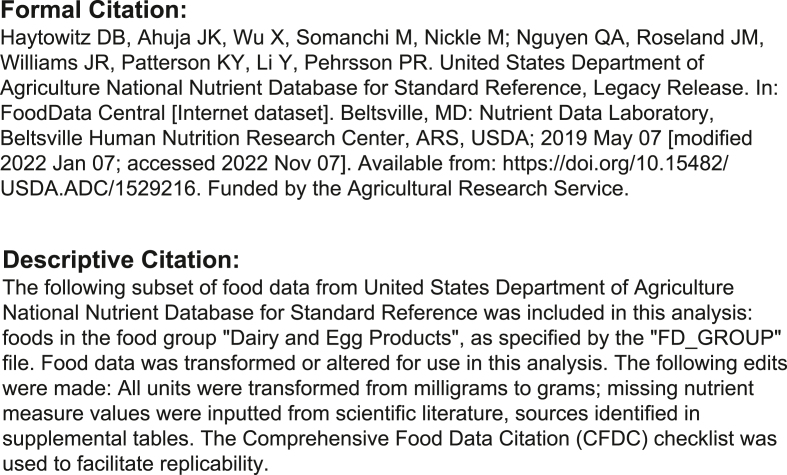
Citation example. Example of formal and descriptive citation for USDA SR Legacy, created using the CFDC citation generator. CFDC, Comprehensive Food Data Citation.

We acknowledge that, at least initially, executing the CFDC checklist as a standard practice will require authors and journals to dedicate time and effort to adopting this more comprehensive approach, which may seem burdensome or unrealistic. To alleviate the difficulty, a CFDC citation generator is now available for use, and can be found at https://www.nutrientinstitute.org/cfdc. The CFDC citation generator provides both formal citation as well as a suggested descriptive in-text citation. Citation formatting follows the National Library for Medicine Style Guide for Authors, Editors, and Publishers [Internet]. 2nd edition [19]. The added value of the CFDC standard and citation generation tool is the inclusion of all validated citation components paired with standard citation formatting aligned with predominant nutrition journals. Figure 2 shows proposed examples of a formal citation and a descriptive citation created with the CFDC citation generator.

## Discussion

Scientific progress depends on knowing and having access to information that has already been reported, tested, and verified. Data citation standards and practices are essential tools for recording such information and allowing others to have access to it. However, using such standards is not intuitive: research communities themselves must create, institute, teach, and uphold citation practices that preserve necessary data and access thereto. Despite researchers’ best intentions, the lack of best-practices knowledge or clear guidelines can result in datasets that are difficult to reproduce [12] or, at the very least, generate misguided presumptions concerning a study’s findings [20]. The ever-increasing production and dissemination of peer-reviewed and non-peer-reviewed data, as well as the globalization of unverified information, will only augment existing challenges to rigorously reproducing and verifying results [20].

### The need for a comprehensive standard for food and nutrient data citation

The concept of quality citation is not new. In a cleverly entitled paper from 1995, “(Not) Giving Credit Where Credit Is Due: Citation of Data Sets,” Sieber and Trumbo [21] address the vital role of adequate citation to verify and build upon the data or methods of the original researcher. More recently (2012), in “The Anatomy of a Data Citation: Discovery, Reuse and Credit,” Mooney and Newton [17] eloquently state that data citation should be a necessary corollary of data publication and reuse, allowing for the identification, retrieval, replication, and verification of data underlying published studies. This recurring reminder in the literature from a growing number of publications spanning multiple fields of science on the importance of quality citation for facilitating quality science demonstrates the need for more effective solutions [1,20].

Although food composition data are a foundational element of nutritional sciences research, the field currently lacks a standard for citing food and nutrient data in research papers and for ensuring that such data are presented in a way that allows studies to be reproduced. The analysis performed in this study found that all of the 165 citations we analyzed, from papers published in the 5 highest-ranked journals in the field, were deficient in some aspect: citations were unidentifiable because of incomplete information, did not lead to the original source of food composition data used in the analysis, contained contradictory (and therefore inaccurate) information, or did not provide minimally complete information. Such findings strongly indicate that nutrition science needs citation standards. To help resolve this data citation quality problem with respect to nutrition research, the proposed CFDC checklist incorporates established data citation components from the data sciences and biological sciences. Representing the highest data citation standards from their respective fields, these components thus constitute the most comprehensive criteria possible for establishing the first and best practices for nutrition sciences research.

Furthermore, the CFDC checklist incorporates data citation guidance beyond merely listing the citation components. It provides examples for both formal (standard) and in-text (descriptive) citations because each type of citation conveys important information. The CFDC checklist therefore has 2 sections: formal citation components and descriptive citation components. Formal citation components are used to locate the data source, and we propose that they should be cited in the formal reference section of the manuscript. For example, version is included because databases are always being updated: the version that an author uses for an analysis could be obsolete and/or changed significantly within a short period of time. For instance, FoodData Central [22] publishes new downloadable versions of their data twice a year. With each new release, new foods are added, foods may be removed or recategorized, and nutrient content of foods can be expanded, removed, or altered. Therefore, knowing the version used will avoid unnecessary searches for old data that may no longer exist in a new database. Other fundamental components of the formal citation are outlined in Table 1.

Descriptive citation components, when applicable, include how the data were used or modified by the author(s). We propose that these descriptive components should be cited in the text of the manuscript. The additional details of edits made to data cited, for example, are crucial for tracing manipulated data in research. With no malfeasance intended, authors may adjust, adapt, or cut out data for analyses but do not record these details. Without such records, it will be very difficult to verify the information published.

The goal of creating the CFDC checklist, with both formal and descriptive citation components, is to enable researchers to have access to data that are as accurate and complete as possible. Meaningful scientific progress is not possible without accurate and complete data. We, therefore, make 3 recommendations. First, all food composition data sources used should be cited using the CFDC checklist as a guide, including specific data citation information that may go beyond the data provider’s or journal’s recommendations.

Second, the formal citation should be included in the references section of the manuscript and additional descriptive citation components provided in-text or in supplemental materials. Finally, where appropriate, authors should indicate if a citation component is not applicable or not provided.

Limitations of the review applied herein may include the fact that only 5 journals were searched, 3 of which were review journals, covering a period of 1 year. Journal selection included top cited journals as ranked by Scimago; we did not evaluate publications from journals that may have ranked differently in other ranking systems. Although the search strategy encompassed most synonyms describing the subject, the lack of standardized terminology means that some articles of interest may not have appeared in the search. In addition, citations that led to a publication may have cited the appropriate food composition data. However, indirect citation is not considered to be the optimal approach.

## Conclusions and perspectives

In this review of food and nutrient data citations, we identified a vital need for implementing a comprehensive standard for data citation in the nutrition sciences. Although these results are not all-inclusive, and certainly do not reflect on the character of the authors or editorial teams of these journals, they point to a pervasive lack of data citation guidance in the nutritional sciences. The CFDC citation checklist created here intends to provide a simple solution to a big problem. The success of this movement falls upon both the data providers and users, and reviewers and editors. To eliminate deficiency in food composition data citation, authors and journals should adopt a rigorous data citation standard, facilitating transparency in data and ability to reproduce research findings. With thousands of nutrition research articles being published every year, steps must be taken to ensure that food composition data are cited consistently and accurately.

## Author contributions

The authors’ responsibilities were as follows – SF: project conception and development of overall research plan; EJ-D: designed research data analysis plan and performed statistical analysis; SF, EJ-D, BB-F: wrote the paper; SF, BB-F: were responsible for study oversight; SF: had primary responsibility for final content; and all authors: read and approved the final manuscript.

## Funding

This work was supported by the Nutrient Institute, a 501(c)(3) not-for-profit organization.

## Data availability

The data that support the findings of this study are available on request from the corresponding author.

## Conflict of interest

BB-F is a Scientific Advisory Board member, SF is Executive Director, and EJ-D is a contractor of the Nutrient Institute 501(c)(3).

## Appendix A Supplementary data

Multimedia component1 Multimedia component1
