# Supplementary material for: Development of a Comprehensive Food Data Citation Standard: A Surprising Gap in the Nutrition Research Literature
Source: Curr Dev Nutr. 2023 Nov 24;8(1):102048. doi: 10.1016/j.cdnut.2023.102048 (PMC10751823; doi:10.1016/j.cdnut.2023.102048)
Supplement: Multimedia component1 [file mmc1.docx]

**Supplemental Table 1.** Report of all data citation frameworks identified

| **Citation Guideline** | **Year Initiated** | **Citation Components** | **Reference Number** |
| --- | --- | --- | --- |
| Data Citation Adequacy Index (DCAI) | 2012 | Author Title Date (of publication) Publisher Material designator URL Persistent Identifier | [(1)](https://paperpile.com/c/LQHkOQ/z7iw) |
| Best Practice Self-Assessment Tool | 2019 | DI BP 1: Persistent data identifiers are associated with all data products DI BP 2: Guidance is provided by an observatory for data citations DI BP 3: Data identifiers are maintained throughout the life cycle of the data, including DI when observatory data are transferred to data aggregators DI BP 4: Data versioning and provenance information is available and accessible. DI BP 5: Processes are in place to track and report data usage DI BP 6: Data usage and citation tracking metrics are provided to the funding agency/stakeholder community | [(2)](https://paperpile.com/c/LQHkOQ/1gqQA) |
| FAIR Data Maturity Model | 2020 | To be Findable: F1. (meta)data are assigned a globally unique and persistent identifier F2. data are described with rich metadata (defined by R1 below) F3. metadata clearly and explicitly include the identifier of the data it describes F4. (meta)data are registered or indexed in a searchable resource  To be Accessible: A1. (meta)data are retrievable by their identifier using a standardized communications protocol A1.1 the protocol is open, free, and universally implementable A1.2 the protocol allows for an authentication and authorization procedure, where necessary A2. metadata are accessible, even when the data are no longer available  To be Interoperable: I1. (meta)data use a formal, accessible, shared, and broadly applicable language for knowledge representation. I2. (meta)data use vocabularies that follow FAIR principles I3. (meta)data include qualified references to other (meta)data  To be Reusable: R1. meta(data) are richly described with a plurality of accurate and relevant attributes R1.1. (meta)data are released with a clear and accessible data usage license R1.2. (meta)data are associated with detailed provenance R1.3. (meta)data meet domain-relevant community standards | [(3)](https://paperpile.com/c/LQHkOQ/mzizV) |
| Computing Index of Citation Adequacy | 1995 | (1) name of the principal investigator (PI) who initially gathered the data (2) title of the data set (3) data of the survey or first release of the data set (4) producer or distributor of the data set (5) address of the producer or distributor (6) funder of the project (7) whether the data set is machine readable (8) whether a code book accompanies the data set | [(4)](https://paperpile.com/c/LQHkOQ/7Mcrv) |
| Data Citation Synthesis Group: Joint Declaration of Data Citation Principles | 2014 | 1. Importance Data should be considered legitimate, citable products of research. Data citations should be accorded the same importance in the scholarly record as citations of other research objects, such as publications.  2. Credit and Attribution Data citations should facilitate giving scholarly credit and normative and legal attribution to all contributors to the data, recognizing that a single style or mechanism of attribution may not be applicable to all data.  3. Evidence In scholarly literature, whenever and wherever a claim relies upon data, the corresponding data should be cited.  4. Unique Identification A data citation should include a persistent method for identification that is machine actionable, globally unique, and widely used by a community.  5. Access Data citations should facilitate access to the data themselves and to such associated metadata, documentation, code, and other materials, as are necessary for both humans and machines to make informed use of the referenced data.  6. Persistence Unique identifiers, and metadata describing the data, and its disposition, should persist — even beyond the lifespan of the data they describe.  7. Specificity and Verifiability Data citations should facilitate identification of, access to, and verification of the specific data that support a claim. Citations or citation metadata should include information about provenance and fixity sufficient to facilitate verifying that the specific timeslice, version and/or granular portion of data retrieved subsequently is the same as was originally cited.  8. Interoperability and Flexibility Data citation methods should be sufficiently flexible to accommodate the variant practices among communities, but should not differ so much that they compromise interoperability of data citation practices across communities. | [(5)](https://paperpile.com/c/LQHkOQ/AeOuQ) |
| ICPSR |  | Author Title Distributor Date Version Persistent identifier (such as the Digital Object Identifier, Uniform Resource Name URN, or Handle System) | [(6)](https://paperpile.com/c/LQHkOQ/C4S58) |
| Out of Cite, Out of Mind: The Current State of Practice, Policy, and Technology for the Citation of Data  CODATA-ICSTI Task Group on Data Citation Standards and Practices | 2013 | Author - The creator of the data set.  Title - As well as the name of the cited resource itself, this may also include the name of a facility and the titles of the top collection and main parent subcollection (if any) of which the data set is a part.  Publisher - The organization (or repository) either hosting the data or performing quality assurance.  Publication date - Whichever is later: the date the data set was made available, the date all quality assurance procedures were completed, or the date the embargo period (if applicable) expired. In other standards an “Access Date” field is used to document the date the data set was successfully accessed.  Resource type - Examples: “database” or “data set.”  Edition - The level or stage of processing of the data, indicating how raw or refined the data set is.  Version - A number increased when the data changes, such as the result of adding more data points or rerunning a derivation process.  Feature name and URI - The name of an ISO 19101:2002 “feature” (e.g., GridSeries, ProfileSeries) and the URI identifying its standard definition, used to pick out a subset of the data.  Verifier - Information to verify the identity of the content.  Identifier - A resolvable web identifier for the data, according to a persistent scheme. There are several types of persistent identifiers, but the scheme that is gaining the most traction is the Digital Object Identifier (DOI).  Location - A persistent URL or UNF from which the data set is available. Some identifier schemes provide these via an identifier resolver service. | [(7)](https://paperpile.com/c/LQHkOQ/sHaKc) |
| DataCite | 2011 | Creator: Author(s) of the dataset Title: Name of the dataset Publisher (or Distributor): Repository name Publication Year: Date the dataset was released or published Version: If you have multiple versions of a specific dataset, or an updated set  Identifier: Unique identifier. This is often a DOI, but can also be an URN or Handle System. | [(8)](https://paperpile.com/c/LQHkOQ/CdCzu) |
| OECD | 2015 | • author’s surname, initials • year of publication (in parentheses, followed by a comma) • title of the work: - in italics and initial caps for books, journal titles or databases - in roman text, sentence case and quotation marks for parts within a larger work • title of the series and edition (as appropriate) • publisher • place of publication (city) • DOI or URL. | [(9)](https://paperpile.com/c/LQHkOQ/59uOB) |
| A Proposed Standard for the Scholarly Citation of Quantitative Data | 2007 | - Author - Title  - Date (of publication) - Publisher - Material designator - Edition - URL - Persistent Identifier | [(10)](https://paperpile.com/c/LQHkOQ/lJjMI) |
| Dataverse Project | 2007 | - Author(s) - Year - Dataset Title - Global Persistent Identifier - Data Repository or Archive - Version | [(11)](https://paperpile.com/c/LQHkOQ/HdMnZ) |
| Bibliographic references for numeric social science data files: Suggested guidelines | 1979 | - Author - Title - Date (of publication) - Publisher - Location - Funder - Material designator - Notes - Edition - Parent / series | [(12)](https://paperpile.com/c/LQHkOQ/6mB1b) |
| IASSIST | 2012 | Author: Name(s) of each individual or organizational entity responsible for the creation of the dataset.  Date of Publication: Year the dataset was published or disseminated.  Title: Complete title of the dataset, including the edition or version number, if applicable.  Publisher and/or Distributor: Organizational entity that makes the dataset available by archiving, producing, publishing, and/or distributing the dataset.  Electronic Location or Identifier: Web address or unique, persistent, global identifier used to locate the dataset (such as a DOI). Append the date retrieved if the title and locator are not specific to the exact instance of the data you used | [(13)](https://paperpile.com/c/LQHkOQ/q4HuB) |
| ANSI/NISO Z39.29 | 2010 | - Author - Title - Date (of publication) - Publisher - Location - Material designator - URL - Persistent Identifier - Accessed Date | [(14)](https://paperpile.com/c/LQHkOQ/wNtJD) |
| DCC How-to Guides | 2015 | - Author - Publication Date - Title - Edition - Version - Feature name and URI - Resource type - Publisher - Unique numeric fingerprint (UNF) - Identifier - Location (URL) | [(15)](https://paperpile.com/c/LQHkOQ/c3jKl) |
| ESIP Data Citation Guidelines | 2019 | Author or Creator: The people or organizations responsible for the intellectual work to develop a data set. The data creator.   Public Release Date: When the particular version of the data set was first made available for use (and potential citation) by others.   Title: The formal title of the data set. It may also include version or edition information but should be carefully controlled. A better alternative is to track version information independent of the title. Note this is the title of the data set, not the project or a related publication. It is important for the data set to have an identity and title of its own.   Version ID: Careful versioning and documentation of version changes are central to enabling accurate citation. Data stewards need to track and clearly indicate precise versions as part of the citation for any version greater than 1. It may be appropriate to track major and minor versions.   Repository: The name of the entity that holds, archives, publishes, prints, distributes, releases, issues, or produces the data. This property will be used to formulate the citation, so consider the prominence of the role. This may be an appropriate place to recognize a major sponsor of the data.   Resolvable Persistent Identifier: The unique identifier that provides the ability to access the data. Not all data have Persistent Identifiers (PIDs) or can be digitally accessed, so an alternative method to access metadata, such as a URL or a physical address, can be provided instead.   Access Date: Because data can be dynamic and changeable in ways that are not always reflected in release dates and versions, it is important to indicate when online data were accessed. | [(16)](https://paperpile.com/c/LQHkOQ/a0cOP) |

**References:**

1. [Mooney H, Newton M. The Anatomy of a Data Citation: Discovery, Reuse, and Credit. Journal of Librarianship and Scholarly Communication [Internet]. 2012 [cited 2022 Apr 15]; doi:](http://paperpile.com/b/LQHkOQ/z7iw) [10.7710/2162-3309.1035](http://dx.doi.org/10.7710/2162-3309.1035)[.](http://paperpile.com/b/LQHkOQ/z7iw)

2. [Smith LM, Kearney TD, Rutherford C, Yarincik K. Data Identification, Citation and Tracking Best Practices : a white paper from the observatory best practices/lessons learned series. Consortium for Ocean Leadership; 2019. p. 32. Available from:](http://paperpile.com/b/LQHkOQ/1gqQA) <http://dx.doi.org/10.25607/OBP-505>[.](http://paperpile.com/b/LQHkOQ/1gqQA)

3. [FAIR Data Maturity Model Working Group. FAIR Data Maturity Model. Specification and Guidelines. 2020; doi:](http://paperpile.com/b/LQHkOQ/mzizV) [10.15497/rda00050](http://dx.doi.org/10.15497/rda00050)[.](http://paperpile.com/b/LQHkOQ/mzizV)

4. [Sieber JE, Trumbo BE. (Not) giving credit where credit is due: Citation of data sets. Sci Eng Ethics [Internet]. 1995;1:11–20. doi:](http://paperpile.com/b/LQHkOQ/7Mcrv) [10.1007/BF02628694](http://dx.doi.org/10.1007/BF02628694)[.](http://paperpile.com/b/LQHkOQ/7Mcrv)

5. [FORCE11. Data Citation Synthesis Group: Joint Declaration of Data Citation Principles. Martone M, editor. 2014 [cited 2022 Apr 15]; doi:](http://paperpile.com/b/LQHkOQ/AeOuQ) [10.25490/a97f-egyk](http://dx.doi.org/10.25490/a97f-egyk)[.](http://paperpile.com/b/LQHkOQ/AeOuQ)

6. [University of Michigan Institute for Social Research. Citing Data [Internet]. ICPSR. [cited 2022 Apr 15]. Available from:](http://paperpile.com/b/LQHkOQ/C4S58) <https://www.icpsr.umich.edu/web/pages/datamanagement/citations.html>[.](http://paperpile.com/b/LQHkOQ/C4S58) doi: 10.5438/0003.

7. [CODATA-ICSTI Task Group on Data Citation Standards and Practices. Out of cite, out of mind: The current state of practice, policy, and technology for the citation of data. Data Sci J [Internet]. 2013 [cited 2022 Apr 15];12:CIDCR1–CIDCR75. doi:](http://paperpile.com/b/LQHkOQ/sHaKc) [10.2481/dsj.osom13-043](http://dx.doi.org/10.2481/dsj.osom13-043)[.](http://paperpile.com/b/LQHkOQ/sHaKc)

8. [DataCite Metadata Working Group. DataCite Metadata Schema Documentation for the Publication and Citation of Research Data [Internet]. DataCite; 2011 [cited 2022 Apr 15]. Available from:](http://paperpile.com/b/LQHkOQ/CdCzu) <https://schema.datacite.org/archive/kernel-2.1/index.html>[.](http://paperpile.com/b/LQHkOQ/CdCzu)

9. [OECD. Bibliographical referencing: Sources and citations. OECD Style Guide [Internet]. Third Edition. OECD Publishing; 2015 [cited 2022 Apr 15]. p. 56–64. Available from:](http://paperpile.com/b/LQHkOQ/59uOB) <https://play.google.com/store/books/details?id=JkO8CgAAQBAJ>[.](http://paperpile.com/b/LQHkOQ/59uOB) doi: 10.1787/9789264243439-5-en.

10. [Micah A, Gary K. A Proposed Standard for the Scholarly Citation of Quantitative Data [Internet]. D-Lib Magazine; 2007 [cited 2022 Apr 15]. Available from:](http://paperpile.com/b/LQHkOQ/lJjMI) <http://dx.doi.org/10.1045/march2007-altman>[.](http://paperpile.com/b/LQHkOQ/lJjMI)

11. [Data Citation [Internet]. The Dataverse Project. [cited 2022 Apr 15]. Available from:](http://paperpile.com/b/LQHkOQ/HdMnZ) <https://dataverse.org/best-practices/data-citation>[.](http://paperpile.com/b/LQHkOQ/HdMnZ)

12. [Dodd SA. Bibliographic references for numeric social science data files: Suggested guidelines. J Am Soc Inf Sci [Internet]. 1979 [cited 2022 Apr 15];30:77–82. doi:](http://paperpile.com/b/LQHkOQ/6mB1b) [10.1002/asi.4630300203](http://dx.doi.org/10.1002/asi.4630300203)[.](http://paperpile.com/b/LQHkOQ/6mB1b)

13. [DATA CITATION RESOURCES - IASSIST SPECIAL INTEREST GROUP ON DATA CITATION (SIGDC) [Internet]. International Association for Social Science Information Service & Technology. 2012 [cited 2022 Apr 15]. Available from:](http://paperpile.com/b/LQHkOQ/q4HuB) <https://iassistdata.org/community/data-citation-ig/data-citation-resources/>[.](http://paperpile.com/b/LQHkOQ/q4HuB)

14. [ANSI/NISO. ANSI/NISO Z39.29-2005 (R2010) Bibliographic References [Internet]. 2010. Available from:](http://paperpile.com/b/LQHkOQ/wNtJD) <http://dx.doi.org/10.3789/ansi.niso.z39.29-2005R2010>[.](http://paperpile.com/b/LQHkOQ/wNtJD)

15. [Ball A, Duke M. How to cite datasets and link to publications. DCC How-to Guides [Internet]. Digital Curation Centre; 2011 [cited 2022 Apr 15]. Available from:](http://paperpile.com/b/LQHkOQ/c3jKl) <https://www.dcc.ac.uk/guidance/how-guides/cite-datasets>[.](http://paperpile.com/b/LQHkOQ/c3jKl)

16. [ESIP Data Preservation and Stewardship Committee. Data Citation Guidelines for Earth Science Data Version 2 [Internet]. Figshare. 2019 [cited 2022 Apr 15]. Available from:](http://paperpile.com/b/LQHkOQ/a0cOP) <https://esip.figshare.com/articles/online_resource/Data_Citation_Guidelines_for_Earth_Science_Data_Version_2/8441816>[.](http://paperpile.com/b/LQHkOQ/a0cOP) doi: 10.6084/m9.figshare.8441816.
